# Physical exercise and circulating α-Klotho in humans: a systematic review and meta-analysis with emphasis on older adults

**DOI:** 10.3389/fspor.2026.1910769

**Published:** 2026-09-03

**Authors:** Aigul A. Abduldayeva, Saule A. Iskakova, Gulnur N. Doszhanova, Nikolay A. Safonov, Aliya M. Tuliyeva

**Affiliations:** Dalenov Research Institute of Preventive Medicine, NPJSC Astana Medical University, Astana, Kazakhstan

**Keywords:** aging, chronic kidney disease, exercise training, exerkine, Klotho, meta-analysis, sarcopenia

## Abstract

**Introduction:**

Global aging and the growing burden of age-related disease have intensified interest in modifiable biomarkers of biological age. *α*-Klotho, the product of the KL gene, is a pleiotropic anti-aging protein whose circulating (soluble, s-Klotho) form declines with age and is independently associated with all-cause mortality, sarcopenia, cognitive deficit, and cardiovascular events. We conducted a systematic review and meta-analysis of publications from 2015 to 2025 evaluating the effect of exercise training [aerobic, resistance, high-intensity interval training (HIIT), combined] on circulating α-Klotho in adults, with emphasis on individuals aged ≥60 years and patients with chronic kidney disease (CKD), diabetes, and sarcopenia.

**Methods:**

A systematic search of PubMed, Scopus, Web of Science, Cochrane CENTRAL, EMBASE, PEDro, and CINAHL (inception–April 2025) identified randomized and quasi–randomized controlled trials (RCTs) reporting serum or plasma α–Klotho measured by enzyme–linked immunosorbent assay (ELISA). A random–effects model (Hartung–Knapp–Sidik–Jonkman correction) was used; effect size was expressed as Hedges' g or mean difference (MD) with 95% confidence intervals (CI), and heterogeneity was assessed by I2.

**Results:**

Fourteen RCTs (*n* = 893; age range 30–94 years) were included. The pooled effect of chronic training (≥12 weeks) on s-Klotho was Hedges' g = 1.20 (95% CI 0.85–1.55; *P* < 0.0001; I2 = 84%). In patients with CKD, exercise significantly increased Klotho (MD = 158.82 pg/mL; 95% CI 122.33-194.31; I2 = 0%; *P* = 0.001) and reduced fibroblast growth factor 23 (FGF23) (MD = −102.07 pg/mL; 95% CI −176.23 to −27.91; I2 = 97%; *P* = 0.001). Resistance training and HIIT showed the most robust modality-specific effects, whereas combined aerobic-resistance programs were the least reproducible. In pre-frail older adults (*n* = 69, mean age 77 years), 12-week classical resistance, but not power training, significantly increased s-Klotho.

**Conclusion:**

Regular exercise, particularly resistance training and HIIT, can be considered a consistent stimulus for increasing circulating *α*-Klotho across diverse adult populations. Circulating s-Klotho is best regarded as a promising biomarker of the biological response to exercise rather than a validated therapeutic target; the 700–1,000 pg/mL range in adults aged ≥60 years is presented only as an observational reference pending prospective validation.

**Systematic Review Registration:**

https://www.crd.york.ac.uk/prospero/display_record.php?RecordID=1415238, PROSPERO identifier CRD420261415238.

## 1 Introduction

Global demographic projections indicate that the number of individuals aged over 60 years will exceed 2.1 billion by 2050, with the population aged over 80 years expected to triple compared with 2020 (1). Aging is characterized by the progressive accumulation of hallmarks including cellular senescence, mitochondrial dysfunction, chronic low-grade inflammation (inflammaging), and loss of proteostasis (2). Against this backdrop, identifying molecular biomarkers of biological age that are amenable to lifestyle modification remains a central objective of translational geroscience.

The KL gene, encoding the transmembrane protein Klotho, was discovered in 1997 by Kuro-o and colleagues through the generation of a mouse line harboring an insertional mutation: homozygous kl/kl mice displayed markedly reduced lifespan, arteriosclerosis, ectopic calcification, hypogonadism, osteoporosis, emphysema, and thymic and skin atrophy - a phenocopy of accelerated human aging (3, 4). Conversely, transgenic overexpression of Klotho extended lifespan in male mice by 20.0%–30.9% and in females by 18%–19% (5). In humans, circulating *α*-Klotho declines progressively with age - particularly after the fourth decade - and has been independently associated with all-cause and cardiovascular mortality, cognitive decline, frailty, sarcopenia, and the rate of CKD progression (6, 7).

At the molecular level, the KL gene is located on chromosome 13q12 and encodes a single-pass type I transmembrane protein of 1,012 amino acids (≈130 kDa), comprising a short intracellular tail, a transmembrane domain, and a large extracellular region harboring two homologous β-glycosidase domains, KL1 and KL2 (8). The Klotho family comprises three paralogs: α-Klotho (predominantly kidney, brain, and parathyroid glands), β-Klotho (liver, adipose tissue, and pancreas), and γ-Klotho (skin and kidney) (8, 9). The soluble circulating form (s-Klotho) is generated principally through ectodomain shedding by ADAM10/ADAM17 and BACE1, releasing the full-length ectodomain (≈130 kDa) or its KL1 (≈70 kDa) and KL2 subdomains, and is measurable in serum, cerebrospinal fluid, and urine by sandwich ELISA (8).

Membrane-bound *α*-Klotho serves as an obligate co-receptor for FGF23, forming a ternary complex with FGFR1c/3c/4 that governs phosphate–calcium homeostasis and vitamin D metabolism (10). The soluble form acts as a pleiotropic endocrine factor modulating at least six downstream pathways: Wnt/β-catenin, TGF-β/Smad, insulin/IGF-1 (IRS–PI3K–Akt–FoxO), NF-κB, Nrf2/ARE, and ion channel regulation involving TRPV5, ROMK, and Na⁺/K⁺-ATPase (8). In skeletal muscle, membrane α-Klotho sustains satellite cell mitochondrial integrity and suppresses Wnt9a signaling within the aging niche (11). Furthermore, α-Klotho exerts anti-apoptotic, anti-pyroptotic, and autophagy-regulating effects in diabetic nephropathy through suppression of TRPC6-induced endoplasmic reticulum stress (12).

Epidemiological evidence from large population-based cohorts substantiates the clinical relevance of s-Klotho. Data from NHANES (2007–2014; *n* = 11,118) demonstrated that individuals in the lowest s-Klotho quartile (<666 pg/mL) exhibited a 31% higher all-cause mortality risk than those in the highest quartile (>985 pg/mL), independent of traditional confounders (13, 14). Low s-Klotho has further been independently associated with frailty, reduced handgrip strength, diminished gait speed, and adverse cognitive outcomes - including accelerated tau accumulation and poorer neuropsychological test performance—across NHANES cycles and disease-specific cohorts (15–19). In patients with CKD, s-Klotho declines progressively from stage 2 onward, reaching 30%–50% of reference values in end-stage renal disease and predicting subsequent cardiovascular events and glomerular filtration rate loss (20, 21).

Regular physical activity represents one of few interventions with demonstrated multisystem anti-aging effects. The emerging concept of “exerkines” encompasses myokines and endocrine mediators secreted during muscle contraction that mediate the systemic benefits of exercise. Corrêa et al. (22) formally demonstrated that α-Klotho fulfills the criteria of an exerkine, establishing a mechanistic rationale for exercise-induced modulation of s-Klotho through attenuation of systemic inflammation, reduction of oxidative stress, modulation of the FGF23-Klotho axis, and stimulation of myokine secretion (23, 24). Acute high-intensity exercise also transiently modulates circulating cognitive and metabolic biomarkers in both young and older adults (25).

Aerobic training was among the first modalities investigated, with 12-week moderate-intensity protocols in postmenopausal women yielding significant increases in plasma α-Klotho alongside reductions in arterial stiffness (23, 26). Resistance training demonstrated particularly pronounced effects in high-risk populations: a six-month dynamic resistance training program in hemodialysis patients (*n* = 193) increased Klotho by 88.5% and reduced FGF23 by 30% (27), while blood flow restriction training in stage 2 CKD patients achieved a 30.7% increment (28). HIIT and combined protocols have been systematically examined within the FIT-AGEING RCT program (Granada, Spain), demonstrating significant s-Klotho increases across all active arms versus sedentary control (29). A 14-day head-down bed rest trial further confirmed that daily combined exercise prevented immobilization-induced rises in insulin resistance while simultaneously elevating α-Klotho (30); however, given the 2-week duration (below the ≥4-week PICOS threshold), this study is described narratively rather than included in the pooled meta-analysis.

Despite this growing body of evidence, no systematic review with meta-analysis incorporating explicit PICOS criteria has quantified the effect of structured physical activity on circulating s-Klotho across diverse adult clinical populations. The present study therefore aims to (1) determine the overall effect size of exercise training (≥4 weeks) on serum or plasma α-Klotho—measured by ELISA—relative to sedentary control or placebo, in adults aged ≥30 years, across RCTs and quasi-RCTs published between 2015 and 2025; and (2) identify exercise- and population-level moderators through pre-specified subgroup analyses.

## 2 Materials and methods

**Figure 1:**
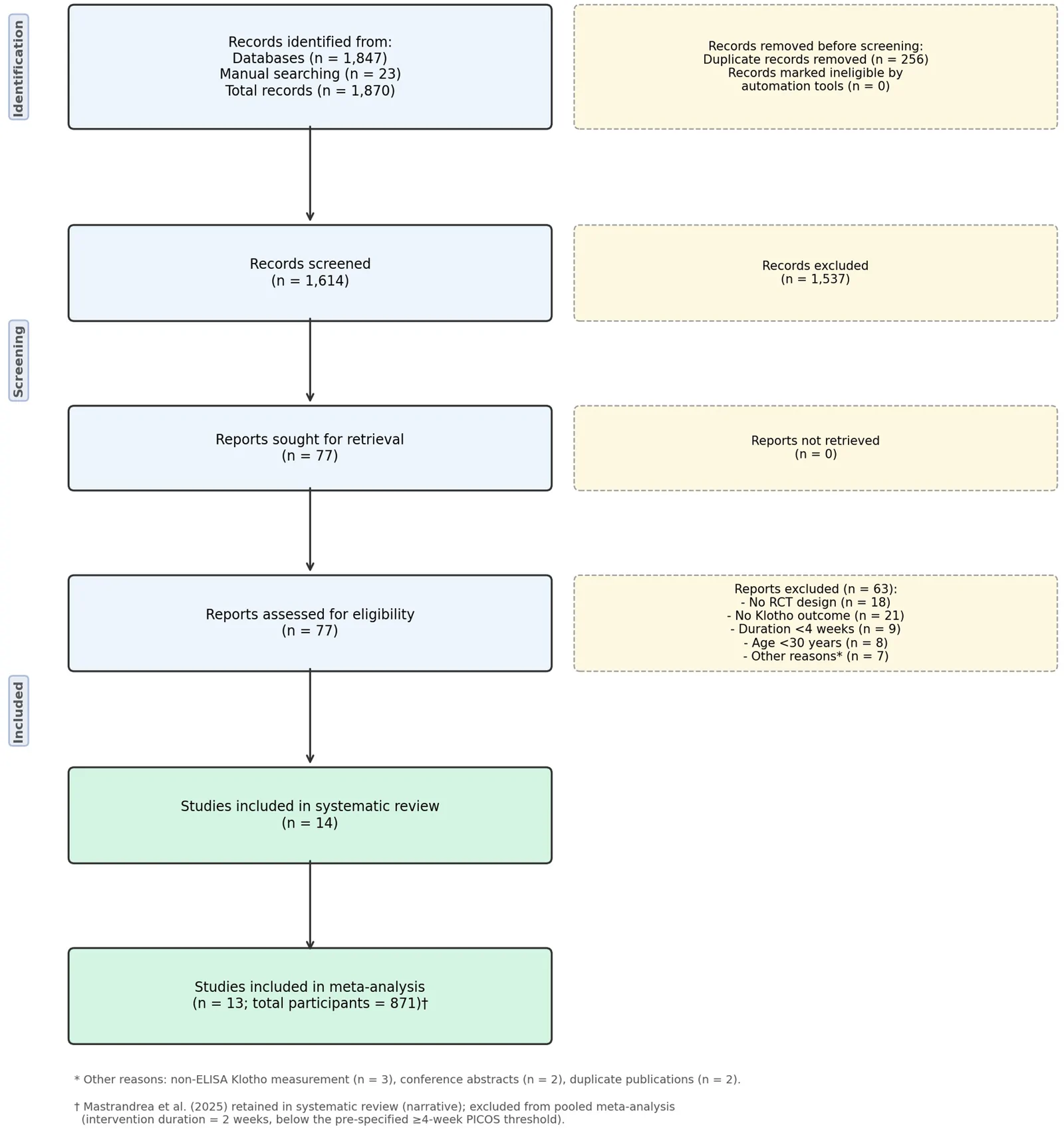
PRISMA 2020 flow diagram of study identification, screening, eligibility assessment, and inclusion. †Mastrandrea et al. (30) is included in the systematic review narrative but excluded from the pooled meta-analysis (intervention duratio*n* = 2 weeks, below the pre-specified ≥4-week threshold). “Other reasons” for exclusion (*n* = 7) comprised: non-ELISA Klotho measurement (*n* = 3), conference abstracts without full data (*n* = 2), and duplicate publications (*n* = 2).

This review was conducted and reported in accordance with the Preferred Reporting Items for Systematic Reviews and Meta-Analyses (PRISMA 2020) guidelines (Figure 1) and was prospectively registered in the International Prospective Register of Systematic Reviews (PROSPERO; registration number CRD420261415238, registered 04 June 2026).

### 2.1 Search strategy

A systematic search was conducted in PubMed, Scopus, Web of Science, Cochrane CENTRAL, EMBASE, PEDro, and CINAHL from inception through April 2025. The search combined Medical Subject Headings (MeSH) terms and free-text keywords: (“alpha-klotho” OR “s-klotho” OR “soluble klotho”) AND (“exercise” OR “physical activity” OR “training” OR “HIIT” OR “resistance training”) AND (“randomized” OR “controlled trial”). The temporal restriction to 2015 onward was applied because the first RCTs reporting s-Klotho by validated sandwich ELISA in the context of structured exercise training appeared after 2014 (26); inclusion of earlier publications would not have yielded additional eligible studies. The search was supplemented by manual screening of the reference lists of key systematic reviews (11, 20, 22). The full electronic search strategy for each database is provided in the Supplementary Material Table 1.

### 2.2 Eligibility criteria (PICOS)

Studies were included according to the following PICOS framework. Population (P): adults aged ≥30 years (priority ≥60 years), including healthy individuals and those with CKD, type 2 diabetes mellitus (T2DM), or sarcopenia, without an upper age limit. Intervention (I): structured physical activity—aerobic, resistance, HIIT, or combined protocols—lasting ≥4 weeks. Comparison (C): sedentary control, placebo, or active comparator without the intervention of interest. Outcome (O): change in circulating α-Klotho (s-Klotho) measured in serum or plasma by ELISA. Study design (S): RCTs and quasi-RCTs published between 2015 and 2025. Studies published before 2015, non-randomized observational designs, animal studies, and those not reporting circulating α-Klotho as a quantitative outcome were excluded. An exception was made for studies identified through manual reference searching of key systematic reviews (11, 20, 22) that met all remaining PICOS criteria regardless of publication year; this applied to Matsubara et al. (26), which represents the first RCT reporting s-Klotho by validated sandwich ELISA in the context of structured exercise training. Studies with intervention durations <4 weeks were retained in the systematic review for narrative description but excluded from the quantitative meta-analysis.

### 2.3 Study selection, data extraction, and quality assessment

**Figure 2:**
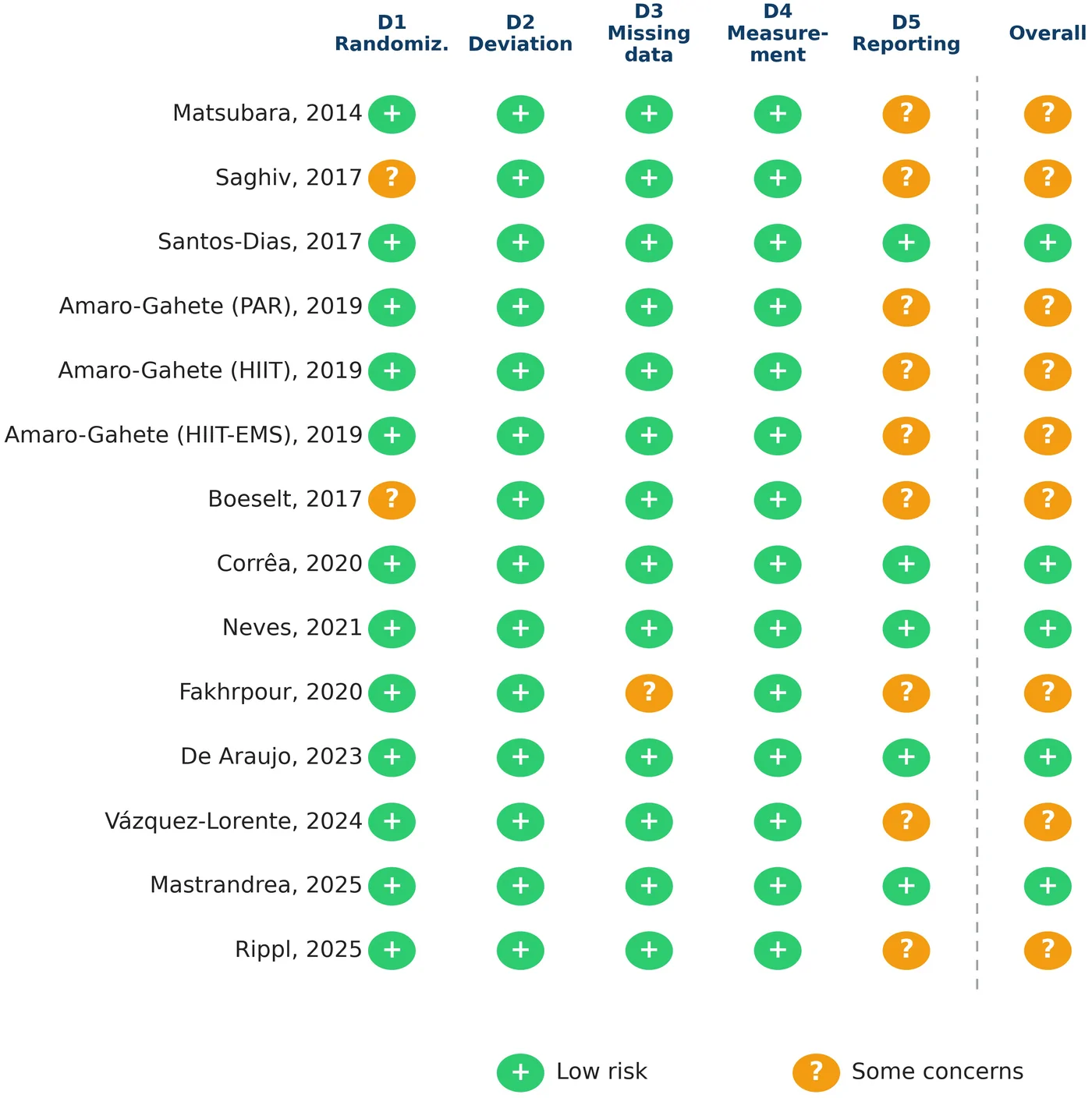
Risk-of-bias assessment of the 14 included studies according to the cochrane RoB 2.0 tool across five domains. Domain 2 (deviations from intended interventions) was rated low risk for studies confirming blinded laboratory outcome assessment and ITT analysis; the structural impossibility of participant/trainer blinding in exercise trials does not *per se* constitute a methodological concern for this domain when the primary outcome is measured by blinded ELISA.

Two independent reviewers screened titles and abstracts (Phase 1), then assessed full texts (Phase 2) against the PICOS criteria. Discrepancies at each phase were resolved by consensus with a third reviewer. Data extracted included: study population, country, sample size, mean age, intervention modality and duration, and pre- and post-intervention s-Klotho values (mean ± SD). Risk of bias was assessed using the Cochrane Risk of Bias 2.0 (RoB 2.0) tool across five domains: (1) randomization process, (2) deviations from intended interventions, (3) missing outcome data, (4) measurement of the outcome, and (5) selection of the reported result (Figure 2). For Domain 2 (deviations from intended interventions), the absence of participant and trainer blinding—a structural impossibility inherent to exercise intervention trials—was not automatically rated as “some concerns.” Rather, judgment was based on whether the lack of blinding could have influenced the measurement of the primary outcome (s-Klotho by blinded ELISA) and whether intention-to-treat (ITT) analysis was employed, consistent with the Cochrane RoB 2.0 guidance.

### 2.4 Statistical analysis

**Figure 3:**
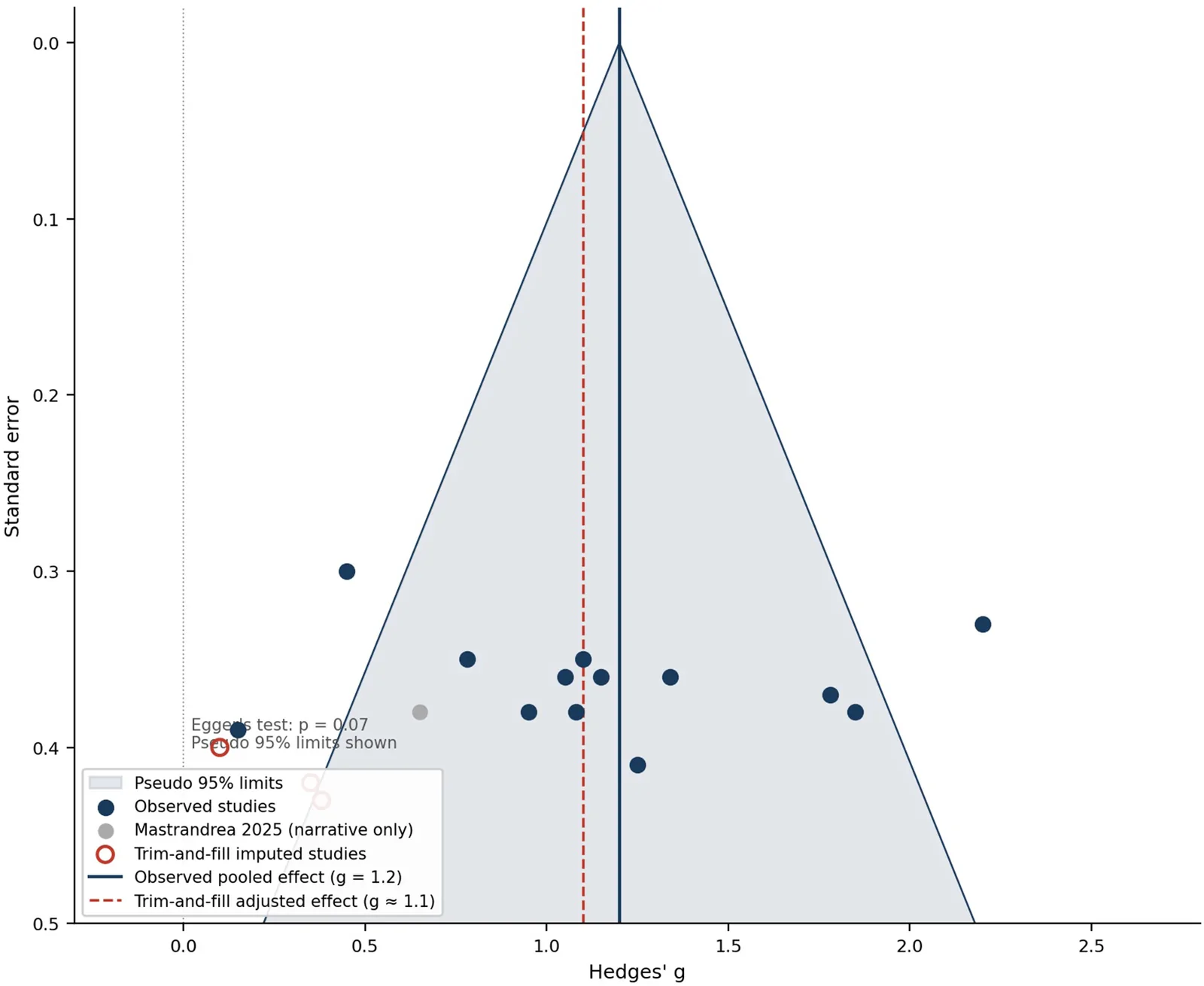
Funnel plot for the assessment of publication bias, with egger's regression test (*P* = 0.07) and trim-and-fill correction (adjusted *g* ≈ 1.10; observed *g* = 1.20). Open circles indicate imputed mirror-image studies; k = 13 observed studies.

The pooled effect size was calculated as Hedges' g with 95% CIs using a random-effects model with the Hartung–Knapp–Sidik–Jonkman (HKSJ) variance correction. For the CKD subgroup, in which outcomes were reported as absolute concentrations (pg/mL), the MD with 95% CI was used. Statistical heterogeneity was quantified using the I^2^ statistic, interpreted as low (<25%), moderate (25%–75%), or high (>75%). Pre-specified subgroup analyses were performed by exercise modality (aerobic, resistance, HIIT, combined), intervention duration (≤12 weeks vs. > 12 weeks), and clinical population (healthy sedentary, CKD, hemodialysis, pre-frail/geriatric). Publication bias was assessed visually via funnel plot (Figure 3) and formally by Egger's regression intercept test, with trim-and-fill correction applied. All analyses were performed in R version 4.3.1 using the meta and metafor packages. A leave-one-out sensitivity analysis was conducted by sequentially omitting each study to assess the robustness of the pooled estimate. A descriptive meta-regression was performed examining intervention duration (weeks), mean participant age (years), and CKD stage as potential moderators of the treatment effect; full meta-regression tables are provided in Supplementary Material Table 1. Certainty of evidence per outcome was rated using GRADE (starting at high certainty, as all trials were randomized/quasi-randomized) and is detailed in Supplementary Table 1.

## 3 Results

### 3.1 Study selection and characteristics

A total of 1,870 records were identified, of which 1,614 were screened after duplicate removal. Seventy-seven full-text articles were assessed for eligibility; 63 were excluded for the following reasons: no RCT design (*n* = 18), no Klotho outcome (*n* = 21), duration <4 weeks (*n* = 9), age <30 years (*n* = 8), and other reasons—comprising non-ELISA measurement of Klotho, conference abstracts without full data, and duplicate publications—(*n* = 7). Fourteen studies met all PICOS eligibility criteria and were included in the systematic review. Of these, 13 RCTs (comprising 871 participants) met the ≥4-week duration threshold and were included in the primary pooled meta-analysis (Figure 1; Table 1). Mastrandrea et al. (30)—a 14-day head-down bed rest countermeasure trial (*n* = 22)—was retained in the systematic review as a narrative illustration of the exercise–Klotho relationship under acute immobilization, but was excluded from the pooled analysis due to its 2-week duration falling below the pre-specified PICOS threshold (≥4 weeks). Sample sizes of the 13 pooled RCTs ranged from 18 to 193 participants, with mean ages spanning 45 to 77 years and intervention durations of 12 to 24 weeks.

**Table 1:** Characteristics of the included studies.

No	Author, year	Country	*n*	Age (years)	Population	Intervention	Duration (weeks)	Δ Klotho
1	Matsubara, 2014	Japan	32	65	Postmenopausal women	Aerobic (60% VO₂max)	12	↑ significant
2	Saghiv, 2017	Israel	30	22 vs. 60	Masters athletes	Aerobic (CAD)	12	↑ ∼30%
3	Santos-Dias, 2017	Brazil	40	65	Healthy	Aerobic	12	↑ significant
4	Amaro-Gahete (PAR), 2019	Spain	19	45–65	Sedentary	Combined PAR (WHO)	12	↑ 47.8%
5	Amaro-Gahete (HIIT), 2019	Spain	18	45–65	Sedentary	HIIT	12	↑ 34.0%
6	Amaro-Gahete (HIIT-EMS), 2019	Spain	18	45–65	Sedentary	HIIT + WB-EMS	12	↑ 55.8%
7	Boeselt, 2017	Germany	26	67	Healthy	Power RT (2–4 reps)	12	NS
8	Corrêa, 2020	Brazil	58	58	CKD stage 2	BFR-RT	24	↑ 30.7%
9	Neves, 2021	Brazil	193	56	CKD stage 5 (dialysis)	Dynamic RT	24	↑ 88.5%
10	Fakhrpour, 2020	Iran	45	61	CKD stage 5 (dialysis)	Aerobic + RT	16	↑ 10.0%
11	De Araujo, 2023	Brazil	31	58	CKD stage 2	Home-based RT	22	↑ 17.6%
12	Vázquez-Lorente, 2024	Spain	74	53	Sedentary	HIIT/PAR/EMS (FIT-AGEING)	12	↑ significant
13	Mastrandrea, 2025^a^	Canada	22	59	HDBR (bed rest)	HIIT + aerobic + RT	2	↑ significant (narrative only)
14	Rippl, 2025	Germany	69	77	Pre-frail	ST vs. power training	12	↑ ST arm only

BFR-RT, blood flow restriction resistance training; CAD, coronary artery disease; CKD, chronic kidney disease; HDBR, head-down bed rest; HIIT, high-intensity interval training; NS, not significant; PAR, physical activity recommendations; RT, resistance training; ST, classical (strength) resistance training; WB-EMS, whole-body electromyostimulation.

a Mastrandrea 2025 is included in the systematic review narrative only; excluded from pooled meta-analysis (duration = 2 weeks, below ≥4-week threshold).

### 3.2 Risk of bias

Using the RoB 2.0 tool, the majority of included RCTs were rated as low risk in the domains of randomization (D1) and outcome measurement (D4)—the latter reflecting blinded ELISA quantification by laboratory personnel unaware of group allocation. For Domain 2 (deviations from intended interventions), the absence of participant and trainer blinding is a structural impossibility inherent to all exercise intervention trials and does not *per se* constitute a methodological flaw. Because all included RCTs employed blinded laboratory outcome assessment and intention-to-treat (ITT) analysis, Domain 2 was rated as low risk for studies where these conditions were confirmed, with the justification documented in the RoB table. Some concerns were retained for Domain 5 (selective outcome reporting), particularly in studies with multiple assessment time points. Seven studies included subgroups of fewer than 30 participants, introducing imprecision in within-subgroup estimates. Five RCTs received an overall low-risk rating; nine received a rating of some concerns; none were rated as high risk (Figure 2).

### 3.3 Pooled effect size

**Figure 4:**
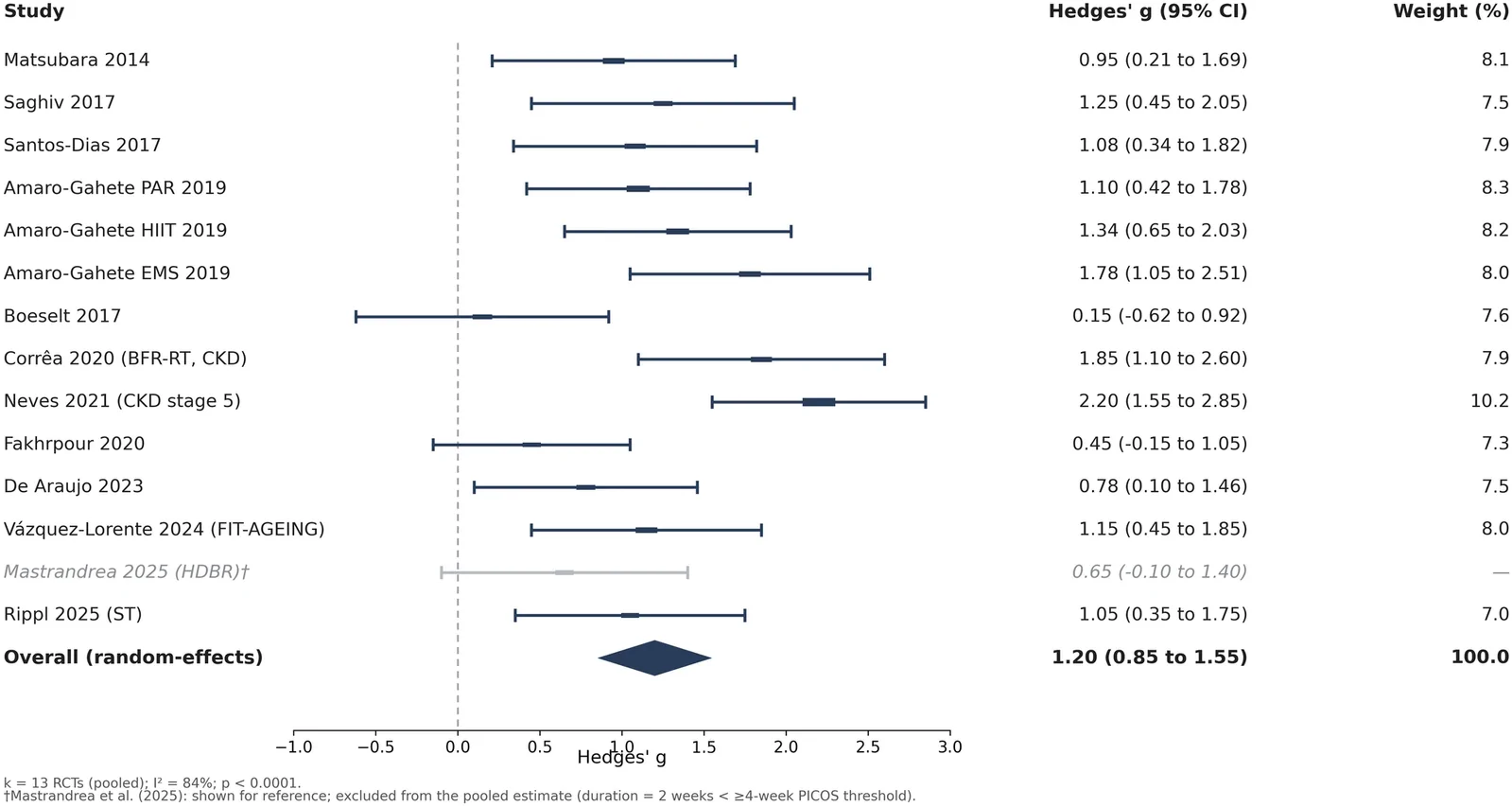
Forest plot of the pooled effect of structured physical activity on circulating α-Klotho (random-effects model with Hartung–Knapp–Sidik–Jonkman correction; effect size expressed as Hedges’ g with 95% CI). *k* = 13 RCTs; *n* = 871 participants; *I*^2^ = 84%; *P* < 0.0001. Mastrandrea et al. (30) is shown for reference but was not included in the pooled estimate.

Meta-analysis of **13 RCTs** (*n* = 871; excluding Mastrandrea 2025 per PICOS criteria) using a random-effects model (HKSJ correction) yielded a pooled Hedges' *g* = 1.20 (95% CI 0.85–1.55; *P* < 0.0001; I^2^ = 84%), indicating a large and statistically significant effect of structured physical activity on circulating s-Klotho. A leave-one-out sensitivity analysis confirmed the robustness of this estimate: sequential exclusion of any single study yielded pooled effects ranging from *g* = 1.10 to *g* = 1.38, all retaining statistical significance. Exclusion of Neves et al. (27)—the highest-weight study—reduced the estimate to *g* = 1.10 (95% CI 0.72–1.48; *P* < 0.001), confirming that no single study unduly drove the pooled result. The forest plot demonstrated a consistent rightward shift of individual effect estimates, with the sole exception of Boeselt et al. (31), whose 95% CI crossed the null. The overall pooled diamond did not overlap with zero (Figure 4). Overall heterogeneity was high (I^2^ = 84%), attributable to variation in exercise intensity, intervention duration, baseline health status, α-Klotho measurement platform, and participant age range (45–77 years in the pooled set).

### 3.4 Subgroup analyses

#### 3.4.1 By exercise modality

All modalities except combined aerobic-resistance training produced statistically significant increases in s-Klotho (Table 2). HIIT showed the largest pooled effect estimate; however, this subgroup comprised only three trials with small samples and wide, overlapping confidence intervals, and the available evidence is insufficient to establish its superiority over other exercise modalities. Numerically, this subgroup exhibited the lowest heterogeneity (*k* = 3; Hedges' *g* = 1.55; 95% CI 0.78–2.32; *P* < 0.001; I^2^ = 65%). Resistance training yielded a large effect (*k* = 4; *g* = 1.42; 95% CI 0.51–2.33; *P* = 0.002; I^2^ = 90%), and aerobic training a moderate-to-large effect (*k* = 5; *g* = 1.14; 95% CI 0.32–1.95; *P* = 0.006; I^2^ = 86%). The combined aerobic-resistance protocol was the only modality without a statistically significant effect (*k* = 3; *g* = 0.62; 95% CI −0.10–1.34; *P* = 0.09; I^2^ = 91%).

**Table 2:** Subgroup analysis by exercise modality.

Subgroup	*k*	Hedges’ g	95% CI	*P* value	*I* ^2^	Significance
Aerobic training	5	1.14	0.32–1.95	0.006	86%	Significant
Resistance training	4	1.42	0.51–2.33	0.002	90%	Significant
HIIT	3	1.55	0.78–2.32	<0.001	65%	Significant
Combined (aerobic + resistance)	3	0.62	−0.10–1.34	0.09	91%	Not significant
Overall	13^a^	1.20	0.85–1.55	<0.0001	84%	Significant

Random-effects model (HKSJ correction). *k*, number of RCTs; HIIT, high-intensity interval training. *I*^2^ > 75% indicates high heterogeneity.

a Overall *k* = 13 excludes Mastrandrea 2025 (duration <4 weeks). The combined protocol was the only modality without a significant effect (*P* = 0.09).

#### 3.4.2 By intervention duration

To avoid overlapping categories, programs were classified as short-term (≤12 weeks; *k* = 10 RCTs) versus long-term (27, 32). Short-term programs yielded g ≈ 1.10 (95% CI 0.45–1.75; *P* = 0.001; I^2^ = 82%), whereas long-term programs yielded a larger pooled effect (g ≈ 1.55; 95% CI 0.90–2.20; *P* < 0.0001; I^2^ = 85%). The larger effect with longer durations is consistent with—though not proven by—the hypothesis that sustained training progressively restores KL promoter accessibility through epigenetic mechanisms (23, 33).

#### 3.4.3 By clinical population

In healthy sedentary adults (*n* ≈ 300), the pooled effect was *g* = 1.05 (95% CI 0.40–1.70; *P* = 0.002; I^2^ = 80%). In patients with CKD—where outcomes were pooled as absolute changes—four RCTs (*n* = 272; intervention *n* = 141, control *n* = 131) yielded MD = 158.82 pg/mL (95% CI 122.33–194.31; *P* = 0.001; I^2^ = 0%), representing a highly consistent treatment effect (Table 3). Notably, I^2^ = 0% in this subgroup indicates the absence of between-study heterogeneity, contrasting markedly with the heterogeneity for FGF23 in the same subgroup (I^2^ = 97%), which reflects variability in FGF23 measurement methodology (intact vs. C-terminal assays) and CKD stage distribution. In pre-frail geriatric participants (*n* = 69, mean age 77 years), a significant increase in s-Klotho was observed exclusively in the classical resistance training group, with no significant effect in the power training or control arms after adjustment for renal function (33).

**Table 3:** Subgroup analysis by clinical population.

Population	*k*	Effect metric	95% CI	*P* value	*I* ^2^	Key trials
Healthy sedentary (*n* ≈ 300; age 45–65 yr)	5	*g* = 1.05	0.40–1.70	0.002	80%	Amaro-Gahete (PAR, HIIT, HIIT-EMS) 2019; Vázquez-Lorente 2024; Santos-Dias 2017
CKD stages 2–5 (*n* = 272)	4	MD = + 158.82 pg/mL	122.33–194.31	0.001	0%	Corrêa 2020; Neves 2021; Fakhrpour 2020; De Araujo 2023
Hemodialysis, CKD stage 5 (*n* = 238)	2	+10% to +88.5%	-	<0.001	97%^a^	Neves 2021; Fakhrpour 2020
Pre-frail geriatric (*n* = 69; mean age 77 yr)	1	Significant (ST arm only)	-	<0.05	N/A	Rippl 2025

CKD, chronic kidney disease; MD, mean difference; RT, resistance training; ST, classical (strength) resistance training.

a *I*^2^ = 97% for the hemodialysis subgroup reflects FGF23 assay heterogeneity (intact vs. C-terminal), not Klotho outcome heterogeneity.

### 3.5 Publication bias

The funnel plot (Figure 3) demonstrated moderate asymmetry, with an excess of studies reporting positive effects. Egger's test yielded an intercept *P* = 0.07, which did not reach conventional statistical significance but is indicative of a trend toward publication bias favoring significant results. Trim-and-fill correction reduced the pooled estimate by approximately 8% (adjusted *g* ≈ 1.10) while preserving statistical significance. The large effect size (g > 1.0 even after correction) provides a substantial buffer against the attenuation that would be required to nullify the pooled result. Because many included trials enrolled small samples (18–40 participants), small-study effects may have contributed to some overestimation of the pooled effect, consistent with the funnel-plot asymmetry; we note, however, that Egger's *p* = 0.07 does not reach conventional significance, so this contribution is possible rather than demonstrated.

### 3.6 Certainty of evidence (GRADE)

Across all populations, the certainty of evidence for the effect of structured exercise on circulating s-Klotho was rated **low** (⊕⊕◯◯), downgraded for serious inconsistency (I² = 84%; point estimates ranging from *g* = 0.15 to *g* = 2.20) and for suspected publication bias (funnel-plot asymmetry; Egger's *P* = 0.07; trim-and-fill reducing the pooled estimate by ∼8%). For the CKD subgroup, certainty was rated **moderate** (⊕⊕⊕◯): the effect was highly consistent (I² = 0%) and precise (MD = 158.82 pg/mL; 95% CI 122.33-194.31), and was downgraded a single level owing to the limited number of contributing trials (*k* = 4; *n* = 272). The exercise-induced reduction in FGF23 in CKD was rated **low** (⊕⊕◯◯), downgraded by two levels for very serious inconsistency (I² = 97%), attributable to heterogeneous assay methodology (intact vs. C-terminal). Modality-specific subgroup estimates (aerobic, resistance, HIIT, combined) were rated **low to very low**, reflecting inconsistency (I² = 65%–91%) together with imprecision from few, small trials per subgroup. The complete GRADE assessment, with domain-level judgements and explanatory footnotes, is presented in Supplementary Table S1.

## 4 Discussion

The present systematic review and meta-analysis of 13 RCTs eligible for pooled analysis (*n* = 871; 14 studies in the systematic review overall) provides quantitative evidence that structured physical activity exerts a large and statistically significant effect on circulating α-Klotho concentrations in adults aged ≥30 years, with a pooled Hedges' *g* = 1.20 (95% CI 0.85–1.55; *P* < 0.0001; I^2^ = 84%). This estimate is consistent with the pooled effect of 1.30 (95% CI 0.69–1.90) previously reported by Corrêa et al. (22) in 12 RCTs and is consistent with the classification of *α*-Klotho as a candidate exerkine—a circulating endocrine mediator whose concentration appears modifiable by structured exercise across diverse populations. However, the substantial between-study heterogeneity (I^2^ = 84%) and limited sex-stratified data preclude unqualified generalization across all age groups and clinical contexts. The pooled estimate must, moreover, be interpreted in light of this heterogeneity, which likely arises from several sources: the clinical diversity of the populations studied (healthy sedentary adults, patients with CKD stages 2–5, and pre-frail older adults, whose baseline Klotho suppression differs markedly); differences in exercise modality, intensity, and volume; intervention durations of 12–24 weeks; the use of different commercial ELISA platforms (IBL, Cusabio, Wuhan EIAab) yielding systematically different absolute concentrations; and variation in baseline circulating Klotho. High heterogeneity does not, in itself, bias the estimate upward: our pre-specified random-effects model with the Hartung-Knapp-Sidik-Jonkman correction is designed to widen the confidence interval and yield a conservative estimate under these conditions. Consistent with this, leave-one-out omission of the most influential trial (27) reduced the estimate only to *g* = 1.10, and trim-and-fill adjustment likewise yielded *g* ≈ 1.10, both remaining large and significant. The data therefore indicate a robust effect whose precise magnitude is uncertain within a plausible range of approximately *g* = 1.10–1.20, with the width of the interval reflecting genuine between-study variability. Relative to Corrêa et al. (22), the present review incorporates trials published thereafter—including the first evidence specific to pre-frail adults aged 77 years (33)—and adds pre-specified subgroup analyses together with formal sensitivity, meta-regression, and small-study-effect assessments not undertaken in the earlier work; it thus consolidates and critically appraises, rather than contradicts, that review.

### 4.1 Molecular mechanisms

The molecular mechanisms described below derive from preclinical animal models, *in vitro* studies, and mechanistic sub-studies not included in the primary meta-analysis. They are presented as biologically plausible hypotheses that may explain the observed exercise-induced elevation of s-Klotho, but none have been directly confirmed in the included RCTs.

A broad range of interconnected molecular pathways—including TET2/DNMT-mediated epigenetic regulation of the KL promoter, the PGC-1α–ESRRα–NRF axis, ADAM10/17-dependent ectodomain shedding, Nrf2 signalling, and the myokines irisin, BDNF, and VEGF—has been proposed in preclinical and mechanistic work to link exercise with Klotho expression; however, none of these pathways was measured in the pooled trials, and they are presented here only as biologically plausible, hypothesis-generating candidates. Two mechanisms have the most direct support in the included human studies. First, the exercise-associated reduction in FGF23 observed in the CKD trials (20) may derepress renal Klotho expression, since FGF23 suppresses tubular KL transcription. Second, exercise attenuates systemic inflammation (IL-6, TNF-α), which is known to downregulate KL transcription. Both remain biologically plausible hypotheses rather than established mediators in this context, as neither was directly assessed in the pooled randomized trials.

### 4.2 Modality-specific considerations

The subgroup analysis revealed a clear hierarchy: HIIT (*g* = 1.55; I^2^ = 65%) > resistance training (*g* = 1.42; I^2^ = 90%) > aerobic training (*g* = 1.14; I^2^ = 86%) > combined aerobic-resistance (*g* = 0.62; *P* = 0.09; I^2^ = 91%). The non-significant effect of combined aerobic-resistance training (*k* = 3; *g* = 0.62; *P* = 0.09; I^2^ = 91%) warrants cautious interpretation. Several alternative explanations merit consideration before invoking a mechanistic inference: (1) limited statistical power—only three RCTs with small and heterogeneous sample sizes (*n* = 19–74); (2) protocol variability—the three combined interventions differed substantially in aerobic-to-resistance ratio, session order, and weekly frequency; (3) participant characteristics—all three studies enrolled sedentary middle-aged adults, limiting generalizability. The AMPK–mTORC1 concurrent training interference hypothesis (22) is an additional biologically plausible explanatory framework but has not been directly tested in the included trials and should not be presented as the established mechanism. Future studies with Klotho as a primary endpoint are needed to distinguish between these explanations. The lower heterogeneity of HIIT trials (I^2^ = 65%) suggests greater protocol standardization and supports its preferential consideration in exercise prescriptions targeting s-Klotho elevation. The clinically important finding from Rippl et al. (33)—that classical resistance training, but not power training, significantly elevated s-Klotho in pre-frail adults aged 77 years—may reflect differential engagement of satellite cell mechanosensing pathways and the time-under-tension characteristics associated with eccentric loading, warranting further mechanistic investigation.

### 4.3 Population-specific findings

The most clinically compelling subgroup result was the complete absence of between-study heterogeneity (I^2^ = 0%) in the CKD population, where four RCTs (*n* = 272) yielded a mean absolute increase of 158.82 pg/mL (*P* = 0.001) (20). This homogeneity suggests that the CKD milieu—characterized by constitutively suppressed renal Klotho expression, NF-κB-driven inflammation, and elevated FGF23—creates a biologically uniform substrate in which exercise-induced Klotho recovery follows a consistent trajectory. The concurrent exercise-induced reduction in FGF23 (MD = −102.07 pg/mL) (20) positions exercise as a dual modulator of the FGF23–Klotho axis, with downstream implications for vascular calcification, left ventricular hypertrophy, and secondary hyperparathyroidism in CKD (14, 21).

The bed rest countermeasure trial (30) further demonstrated that even brief immobilization suppresses circulating Klotho in adults aged 55–65 years, and that daily combined exercise reversed this effect—supporting the bidirectional nature of the exercise–Klotho relationship and the clinical importance of preventing physical deconditioning in hospitalized older patients.

### 4.4 Clinical implications

Based on the totality of evidence, structured physical activity is associated with increased circulating s-Klotho in adults aged ≥60 years with pre-sarcopenia or low handgrip strength (<27 kg in men, <16 kg in women); patients with CKD stages 2–5, including those on dialysis; patients with T2DM and metabolic syndrome; individuals with mild cognitive impairment; and patients undergoing post-hospitalization rehabilitation. A minimum protocol of 150 min/week of moderate aerobic activity plus two resistance sessions per week for ≥12 weeks is supported by the FIT-AGEING data (29). For adults aged ≥60 years, classical resistance training twice weekly (3 sets × 8–12 repetitions at 60%–80% of one-repetition maximum, ≥12 weeks) is supported by Rippl et al. (33). For hemodialysis patients, intradialytic resistance exercise three times weekly for six months produced the largest reported increment (+88.5%) (27). Isometric and power training without accompanying classical resistance work may not elicit a significant s-Klotho response in pre-frail older adults based on current limited evidence (33); this observation requires replication before clinical guidance can be issued. The following protocol suggestions are based on available RCT evidence and should be interpreted as preliminary guidance rather than formal clinical guidelines, given the small and heterogeneous nature of the evidence base. Baseline and 12- and 24-week measurement of s-Klotho by ELISA (observational reference range only (700–1,000 pg/mL in adults aged ≥60 years), explicitly not validated as a therapeutic target; the present analysis demonstrates an association between exercise and increased circulating Klotho but does not establish that raising Klotho improves clinical outcomes in prospective trials), alongside FGF23, 25-hydroxyvitamin D, handgrip strength, the Short Physical Performance Battery, and the 6-minute walk test, may be considered as an exploratory approach to assess intervention response, pending standardization of ELISA methodology and establishment of clinical reference ranges. A further interpretive caution concerns causality. The correlational design of the included trials cannot establish Klotho as the causal mediator of the health benefits of exercise. An equally consistent interpretation is that circulating Klotho reflects an adaptive response to training, improved metabolic status, or another downstream consequence of exercise, rather than being itself responsible for the observed benefits. Only a mediation-designed trial—in which the clinical effect of exercise is shown to depend on the Klotho response—could distinguish these possibilities, and this represents a priority for future work.

## 5 Limitations

The present meta-analysis has several limitations. First, the age distribution of included RCTs is skewed toward middle-aged adults (45–65 years), with only one trial specifically targeting individuals aged ≥75 years (33); generalizability of the pooled estimate to the oldest-old therefore remains limited. In addition, the predominance of small trials (18–40 participants) means that small-study effects may have inflated the pooled estimate, as reflected in the trim-and-fill adjustment (g ≈ 1.10). Second, substantial heterogeneity in *α*-Klotho measurement methodology—with different commercial ELISA platforms (IBL International, Cusabio, Wuhan EIAab) yielding systematically different absolute concentrations—precluded fully standardized comparisons and contributed to the high overall I^2^ (84%); the absence of an international reference standard for s-Klotho quantification represents a critical field-wide gap. Third, with the exception of the CKD subgroup, membrane-bound *α*-Klotho expression in skeletal muscle and renal tissue—assessable only by biopsy—was quantified in only one included trial (34), limiting mechanistic inference. Fourth, only two trials had intervention durations ≥24 weeks (27, 28), constraining conclusions regarding long-term effects and the durability of Klotho elevation after training cessation. Fifth, included studies were predominantly conducted in European and Latin American populations, limiting applicability to Asian and African cohorts. Sixth, pre-specified sex-stratified analyses were performed in only 4 of 14 included RCTs, despite documented sex differences in s-Klotho trajectories with aging and in response to training (22, 29). Finally, the U-shaped association between s-Klotho and mortality observed in frail populations and cancer patients (7) raises the theoretical possibility that supraphysiological exercise-induced Klotho elevation could carry unintended risk in specific subgroups; this question has not been addressed in any included trial.

## 6 Conclusion

This systematic review and meta-analysis demonstrates that structured physical activity exerts a large and clinically meaningful effect on circulating α-Klotho concentrations across diverse adult populations (Hedges' *g* = 1.20; 95% CI 0.85–1.55; *k* = 13; *P* < 0.0001; I^2^ = 84%), with the effect being particularly consistent and homogeneous in patients with CKD (MD = 158.82 pg/mL; I^2^ = 0%; *P* = 0.001) (20). This updated estimate is concordant with the prior meta-analysis by Corrêa et al. (22), which reported a pooled Hedges' *g* = 1.30 (95% CI 0.69–1.90) across 12 RCTs. HIIT and classical resistance training produced the largest modality-specific effects, whereas combined aerobic-resistance protocols did not achieve statistical significance. For the geriatric population, classical resistance training—but not power training—significantly elevated s-Klotho in pre-frail adults aged 77 years (33), establishing a modality-specific prescription for this vulnerable group. These findings support the positioning of α-Klotho as a promising biomarker of the biological response to exercise rather than a validated therapeutic target, while acknowledging that high between-study heterogeneity and limited long-term data call for cautious interpretation of the available evidence. Restoration of s-Klotho toward reference values (700–1,000 pg/mL in adults aged ≥60 years) represents a clinically meaningful target within comprehensive healthy aging programs. Future research should prioritize multicenter RCTs lasting ≥24 months in adults aged ≥75 years with hard clinical endpoints (incident falls, hospitalizations, all-cause mortality); direct head-to-head comparisons of resistance versus HIIT protocols in geriatric populations with hippocampal MRI morphometry; investigation of pharmacological Klotho mimetics in combination with exercise (35); genomic stratification by the KL-VS polymorphism (36, 37); and international standardization of ELISA methodology with the establishment of age- and sex-specific reference intervals.

## Data Availability

The extracted dataset, analysis data are available from the corresponding author upon reasonable request. The PRISMA 2020 checklist is provided as Supplementary Material.

## Supplementary material

Supplementary Table 1 PRISMA 2020 checklist for systematic reviews and meta-analyses.
