# Supplementary material for: Physical exercise and circulating α-Klotho in humans: a systematic review and meta-analysis with emphasis on older adults
Source: Front Sports Act Living. 2026 Sep 3;8:1910769. doi: 10.3389/fspor.2026.1910769 (PMC13582480; doi:10.3389/fspor.2026.1910769)
Supplement: Supplementary file 1 [file Table1.docx]

**PRISMA 2020 Checklist**

Manuscript: “Physical exercise and circulating α-Klotho in humans: a systematic review and meta-analysis with emphasis on older adults”

Journal: Frontiers in Sports and Active Living — Systematic Review article type

*Note: “Location where item is reported” refers to sections of the Frontiers-formatted manuscript. Continuous line numbers are present in the manuscript; insert specific line numbers in this column where your editor requests them.*

| **Section and topic** | **Item #** | **Checklist item** | **Location where item is reported** |
| --- | --- | --- | --- |
| **TITLE** | | | |
| Title | 1 | Identify the report as a systematic review. | **Confirmed.** The title is correct and unchanged: “Physical exercise and circulating α-Klotho in humans: a systematic review and meta-analysis with emphasis on older adults.” No changes required. |
| **ABSTRACT** | | | |
| Abstract | 2 | See the PRISMA 2020 for Abstracts checklist. | Abstract (single structured paragraph) |
| **INTRODUCTION** | | | |
| Rationale | 3 | Describe the rationale for the review in the context of existing knowledge. | Introduction (§1) |
| Objectives | 4 | Provide an explicit statement of the objective(s) or question(s) the review addresses. | Introduction (§1, final paragraph; aims 1–2) |
| **METHODS** | | | |
| Eligibility criteria | 5 | Specify the inclusion and exclusion criteria for the review and how studies were grouped for the syntheses. | Methods §2.2 (PICOS) |
| Information sources | 6 | Specify all databases, registers, websites, organisations, reference lists and other sources searched; specify the date when each source was last searched. | Methods §2.1 (7 databases; inception–April 2025) |
| Search strategy | 7 | Present the full search strategies for all databases, registers and websites, including any filters and limits used. | Methods §2.1; Supplementary Appendix (Full search strategies for all 7 databases). |
| Selection process | 8 | Specify the methods to decide whether a study met the inclusion criteria, including number of reviewers, whether they worked independently, and any automation tools. | Methods §2.3 (two independent reviewers; consensus with a third) |
| Data collection process | 9 | Specify the methods used to collect data from reports, including number of reviewers and independence. | Methods §2.3 |
| Data items | 10a | List and define all outcomes for which data were sought; specify whether all compatible results were sought. | Methods §2.2 (Outcome) and §2.4 |
|  | 10b | List and define all other variables for which data were sought; describe assumptions about missing/unclear information. | Methods §2.3 (population, country, n, age, modality, duration) |
| Study risk of bias assessment | 11 | Specify the methods used to assess risk of bias, including the tool(s), number of reviewers and independence. | Methods §2.3 (Cochrane RoB 2.0, 5 domains) |
| Effect measures | 12 | Specify for each outcome the effect measure(s) used in the synthesis or presentation of results. | Methods §2.4 (Hedges’ g; mean difference) |
| Synthesis methods | 13a | Describe processes used to decide which studies were eligible for each synthesis. | Methods §2.4 (pre-specified subgroups) |
|  | 13b | Describe any methods required to prepare the data for presentation/synthesis (e.g. handling of missing summary statistics, conversions). | Methods §2.4 -Where SDs were not reported, they were approximated from 95% CIs or standard errors using standard formulas. Studies reporting percentage change only were converted to standardised effect sizes using pooled pre-intervention SDs. All studies reported s-Klotho in pg/mL (no unit conversion required). - add handling of missing SDs / data conversions |
|  | 13c | Describe any methods used to tabulate or visually display results of individual studies and syntheses. | Methods §2.4; Table 1, Figure 3 (forest plot) |
|  | 13d | Describe methods used to synthesise results and the rationale; if meta-analysis was done, describe model(s), heterogeneity methods and software. | Methods §2.4 (random-effects, HKSJ; I²; R 4.3.1, meta/metafor) |
|  | 13e | Describe any methods used to explore possible causes of heterogeneity (e.g. subgroup analysis, meta-regression). | Methods §2.4; Results §3.4 (subgroups: modality, duration, population) |
|  | 13f | Describe any sensitivity analyses conducted to assess robustness of the synthesised results. | Methods §2.4 and Results §3.5 (trim-and-fill) |
| Reporting bias assessment | 14 | Describe any methods used to assess risk of bias due to missing results in a synthesis (reporting biases). | Methods §2.4 (funnel plot; Egger’s test) |
| Certainty assessment | 15 | Describe any methods used to assess certainty (confidence) in the body of evidence for an outcome. | **Methods §2.4 - a GRADE assessment has been added to this section.** |
| **RESULTS** | | | |
| Study selection | 16a | Describe the results of the search and selection process, ideally using a flow diagram. | Results §3.1; Figure 1 (PRISMA 2020 flow diagram) |
|  | 16b | Cite studies that might appear to meet the inclusion criteria but were excluded, and explain why. | Results §3.1; Supplementary Appendix (List of excluded full-text studies with reasons). |
| Study characteristics | 17 | Cite each included study and present its characteristics. | Results §3.1; Table 1, **(в Author Queries Response)** |
| Risk of bias in studies | 18 | Present assessments of risk of bias for each included study. | Results §3.2; Figure 2 |
| Results of individual studies | 19 | For all outcomes, present for each study summary statistics and an effect estimate with precision, ideally in tables or plots. | Table 1; Figure 3 (forest plot) |
| Results of syntheses | 20a | For each synthesis, briefly summarise the characteristics and risk of bias among contributing studies. | Results §3.3–3.4 |
|  | 20b | Present results of all statistical syntheses; if meta-analysis was done, present the summary estimate, its precision and heterogeneity; describe direction of effect. | Results §3.3–3.4; Tables 2–3 |
|  | 20c | Present results of all investigations of possible causes of heterogeneity. | Results §3.4 (modality, duration, population subgroups) |
|  | 20d | Present results of all sensitivity analyses. | Results §3.5 (trim-and-fill; adjusted g ≈ 1.10) |
| Reporting biases | 21 | Present assessments of risk of bias due to missing results for each synthesis assessed. | Results §3.5 (funnel-plot asymmetry; Egger’s P = 0.07) |
| Certainty of evidence | 22 | Present assessments of certainty (confidence) in the body of evidence for each outcome assessed. | Results §3.6- Formal GRADE assessment not included in current submission. Overall certainty is judged moderate: RCT evidence downgraded for high heterogeneity (I² = 84%), predominantly middle-aged sample, and imprecision in subgroups. |
| **DISCUSSION** | | | |
| Discussion | 23a | Provide a general interpretation of the results in the context of other evidence. | Discussion §4, §4.1–4.3 |
|  | 23b | Discuss any limitations of the evidence included in the review. | Discussion §4.5 (Limitations) |
|  | 23c | Discuss any limitations of the review processes used. | Discussion §4.5 (ELISA heterogeneity; no reference standard; sex-stratification) |
|  | 23d | Discuss implications of the results for practice, policy, and future research. | Discussion §4.4 (Clinical implications) and §4.6 (Conclusion / future research) |
| **OTHER INFORMATION** | | | |
| Registration and protocol | 24a | Provide registration information including the register name and registration number, or state that the review was not registered. | Methods §2 (PROSPERO; **CRD420261415238, registered 04 June 2026**) |
|  | 24b | Indicate where the review protocol can be accessed, or state that a protocol was not prepared. | Methods §2 (protocol via PROSPERO record) |
|  | 24c | Describe and explain any amendments to information provided at registration or in the protocol. | None / Not applicable (state explicitly if any amendments occurred) |
| Support | 25 | Describe sources of financial or non-financial support, and the role of funders or sponsors. | Funding statement (no financial support received) |
| Competing interests | 26 | Declare any competing interests of review authors. | Conflict of Interest statement |
| Availability of data, code and other materials | 27 | Report which of the following are publicly available and where: data collection forms; extracted data; analysis data; analytic code; other materials. | **Data Availability Statement.** The extracted dataset, analysis data are available from the corresponding author upon reasonable request. The PRISMA 2020 checklist is provided as Supplementary Material |

*Reference: Page MJ, McKenzie JE, Bossuyt PM, Boutron I, Hoffmann TC, Mulrow CD, et al. (2021). The PRISMA 2020 statement: an updated guideline for reporting systematic reviews. BMJ 372, n71. doi: 10.1136/bmj.n71*
